# An epidemiological investigation into the reasons for high bovine tuberculosis incidence in cattle herds of the Burren, Ireland, prior to 2020

**DOI:** 10.1186/s13620-024-00275-y

**Published:** 2024-07-20

**Authors:** Jamie Alexander Tratalos, Jamie Michael Madden, Miriam Casey, Catherine McSweeney, Fidelma Mary Farrell, Simon John More

**Affiliations:** 1 UCD Centre for Veterinary Epidemiology and Risk Analysis, UCD School of Veterinary Medicine, University College Dublin, Belfield, Dublin 4, Ireland; 2 Department of Agriculture, Food and the Marine, Limerick Regional Veterinary Office, Houston Hall, Raheen Industrial Park, Raheen, Limerick, V94 PKF1 Ireland

**Keywords:** Bovine tuberculosis, Burren, Epidemiology, Rainfall, *Meles meles*

## Abstract

Herd-level bovine tuberculosis (bTB) incidence was examined in the Burren, an area in the west of Ireland where herd owners practice distinctive transhumance practices, with upland winter grazing. Prior to the initiation of our study in 2020, bTB incidence had for many years been unusually high in the Burren in comparison with the rest of the country, although the most recent figures have come down to being closer to the national average. Using data from the period prior to 2020, we mapped bTB infection in Burren herds alongside a range of indicators thought to have an association with it - herd size, herd density, herd type, cattle movement, and badger (*Meles meles*) population and control data, as well as rainfall and altitude. We also looked at how summary statistics for these variables differed when Burren herds with a history of bTB were compared to other Burren herds, as well as bTB positive and negative herds from outside the Burren. We found that for many indicators Burren herds would be expected to be low risk when compared to other herds in Ireland. An exception to this was for rainfall: hot spot areas for bTB in the Burren were found in areas of higher rainfall, on average herds in the Burren experienced more rainfall than those outside it, and bTB herds in the Burren experienced higher rainfall than non-bTB herds. Separately, for Burren herds only, a logistic regression model was developed to explain bTB breakdown occurrence using a matched case-control approach. Cases were herds which had experienced a new bTB breakdown between 2015 and 2019 (*n* = 260) and these were matched on herd type and herd size with the same number of herds not experiencing a breakdown during this period. This showed that, of a range of exogenous variables, rainfall was the most strongly associated with herd-level bTB incidence. These results suggest that high levels of exposure to inclement weather, and/or better environmental survival of *Mycobacterium bovis* in the environment, may contribute to high bTB rates in the Burren. However, as rainfall showed a highly aggregated distribution, this relationship may be due to an unmeasured factor correlated with it. Mapping and graphical output suggested that, although herd sizes in the Burren were on average lower than nationally, within the Burren they were higher in areas of higher prevalence, suggesting that mechanisms associated with herd size, such as increased contacts between and within herd, and with wildlife, may also play a role.

## Introduction

The Burren, an area in the West of The Republic of Ireland (henceforth “Ireland”), is known for its uplands characterized by glaciated karst geology and the floral communities associated with it. For many centuries its distinctive ecology has been fostered through transhumance farming practices. Known locally as ‘winterage’, pastures are left ungrazed during the summer and cattle are moved onto them for the winter, typically from lower lying summer pastures [1, 2]. This contrasts with transhumance practices formerly followed elsewhere in Ireland [3, 4], which typically consisted of spending the summer at higher elevations, and also with the common practice of housing cattle indoors during the winter. In recent years, financial grants have been made available to herd owners to encourage the survival of livestock management practices traditional in the Burren, and the Burren Programme has been set up to operate as an agri-environmental programme for conservation and support of the heritage, environment and communities of the area ([2]; https://ec.europa.eu/environment/nature/rbaps/fiche/burren-farming-conservation-programme-bfcp_en.htm; http://burrenprogramme.com). Most of the Burren has been designated as a Natura 2000 area under the EU Habitats Directive [5].

Bovine TB (bTB) is endemic amongst the cattle population of Ireland, and mechanisms of transmission of infection include contact between cattle, within the same herd and from other herds (the latter both across farm boundaries and through trade between herds), contact with infected wildlife populations, especially badgers (*Meles meles*), and, possibly, exposure to the causative organism, *Mycobacterium bovis*, residing in the wider environment [6, 7]. However, the relative contribution of each of these factors is poorly understood and is likely to vary from location to location. A national eradication programme has been in place since 1954, and now consists of annual testing of all herds, restriction of cattle sales from all herds deemed to be bTB positive, and removal through culling of badgers in areas where they are thought to contribute to bTB outbreaks [8–10]. In addition, a badger vaccination programme is currently being rolled out across much of Ireland [11].

At the time that this study was conceived (in 2020), bTB prevalence in Burren cattle was believed to be unusually high relative to the rest of Ireland. The reasons for this have not been determined [12] but a number of contributing factors have been suggested, based on the experiences of DAFM staff working alongside Burren stakeholders. These include: (1) The nutritional quality of winter pasture is often poor and can be low in trace minerals and there is limited shelter available for overwintered cattle. The Burren Programme reports that Burren winterages may not meet the nutritional requirements of suckler cattle from January to March. If animals are not given supplementary feed during this period, they may suffer poor body condition and increased susceptibility to infection. (2) Although the availability of housing varies from herd to herd, animals are often exposed to cold temperatures, rain and wind whilst overwintering, which could contribute to immune suppression, fostering the spread of bTB. (3) There may also be a genetic component. There is believed to be a reluctance amongst Burren herd owners to purchase cattle born outside the Burren area, due to a belief that non-local cattle are less resistant to babesiosis, which is endemic in the Burren (see Zintl et al. [13] for a description of the disease in Ireland). It is therefore possible that Burren cattle may have relatively low genetic variability and potentially a predisposition to bTB infection, given that the genetic make-up of individual cattle may predispose them to bTB [14]. (4) Conversely, cattle movement may also play a role, for those farms that do introduce animals. In broader (Ireland-wide) studies, the introduction of cattle has been found to increase herd-level bTB risk, as it facilitates the introduction of bTB-infected cattle into previously uninfected herds [15, 16]. The movement of cattle from diverse locations through transhumance might also increase the risk of cattle picking up bTB from elsewhere and bringing it into the Burren. (5) Areas of eroded karst geology with extensive underground cave systems and dense hazel scrub, and an observed avoidance of regular paths by badgers [17] may reduce the effectiveness of badger control and surveillance activities. (6) Some farmers in the Burren believe that feral goats are responsible for high rates of bTB in the Burren [12]; testing of 16 culled Burren wild goats in 2016 and 2017 found no evidence of bTB infection (Animal Diseases, Dáil Éireann Debate, Thursday − 19 May 2022, https://www.oireachtas.ie/en/debates/question/2022-05-19/401/.) (7) Finally, it has been hypothesised that high rates of liver fluke infection might be responsible for higher levels of bTB, both through immunosuppressive effects giving rise to poor reliability of the skin test [18] and also through effects on the general health of the animal, leading to increased susceptibility to bTB [19, 20].

In the face of concern surrounding high bTB prevalence in Burren herds and an incomplete understanding of the causes, farming stakeholders in association with the Burren Programme and herd owners in the Burren, requested an epidemiological investigation of bTB in the region. As part of this investigation, this study was undertaken to document the characteristics of Burren herds of relevance to bTB transmission and to investigate the possible causes of high rates of bTB there. This was achieved by comparing Burren herds, with and without bTB versus herds in the rest of Ireland, using data from 2015 to 2019, and also a matched case-control study, exclusively for Burren herds, again using 2015–2019 data. Our study is complementary to a detailed survey questionnaire which was circulated to Burren herd owners, the results of which are reported in Clarke et al. [12].

## Materials and methods

### Herds studied and approach used

**Fig. 1:**
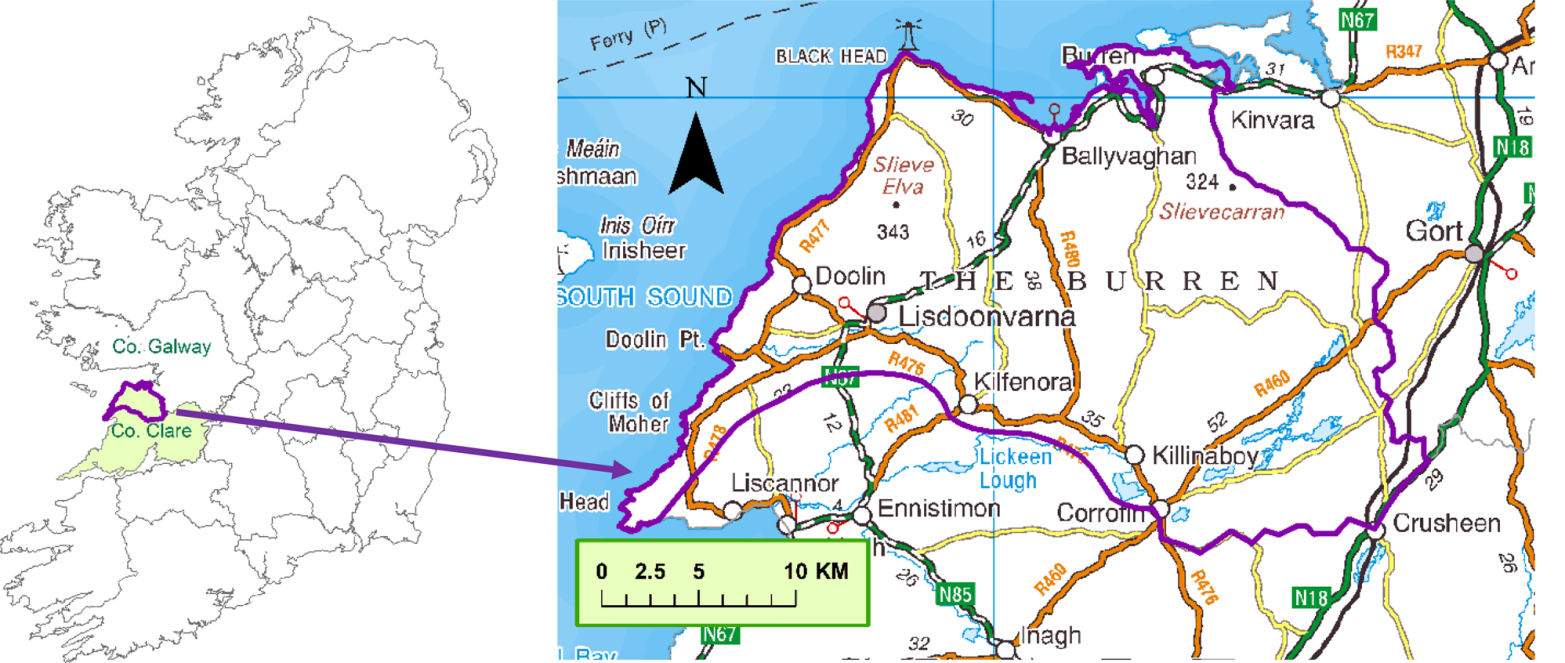
Map of the study area (purple border in both maps). The map on the left shows its position relative to the rest/ of Ireland, with County Clare shaded in green

This study was conducted at the request of the Burren Programme, local herd owners, and veterinary staff of the Department for Agriculture, Food, and the Marine (DAFM). The study area, which was defined in consultation with these stakeholders, consisted of the area of the Burren Geopark (https://www.burrengeopark.ie/) lying within County Clare, in addition to an area to the southeast around Crusheen (Fig. 1). Any herd with a land parcel falling inside the study area was classified as a Burren study herd. Approximately 5% of Irish herds have not been mapped precisely, and these were therefore not available for selection. These analyses consisted of a series of maps, bivariable analyses using bar plots, and the production of a multivariable binary regression model, described in detail below. Some of these analyses were restricted to looking at differences amongst the Burren study herds whereas others in addition compared them to herds outside the study area.

### Data sources

We used the following data sources:

#### bTB testing

The bTB test history of each herd in Ireland, comprising results of the Comparative Intradermal Tuberculin Test (CITT, formally known as the Single Intradermal Comparative Tuberculin Test, SICTT), gamma interferon tests and slaughterhouse inspections, was obtained from DAFM’s Animal Health Computer System (AHCS). Each Irish cattle herd is tested at least once per year. If any cattle in the herd test positive for bTB, the herd is restricted from moving cattle to other herds until further testing is suggestive of freedom from infection (more details can be found in Good et al. 2010 [21]). These data were used to calculate whether a herd tested positive for bTB over a given period, when herds were placed under restriction, and the proportion of skin reactors with visible lesions at slaughter.

#### Herd locations and land parcels

DAFM’s Land Parcel Information System (LPIS), 2018 [22], was used to delineate the extent of the land occupied by each cattle herd. Herd locations were determined as the centroid of the largest land fragment occupied by the farm.

#### Cattle movement data

The births, movement records and end of year herd populations for all Irish cattle during the period 2010 to 2019 were acquired from DAFM’s Animal Identification and Movements (AIM) database. Analyses conducted on movement data from the same source are described in more detail in previous studies [16, 23, 24]. These data were used to estimate movement network variables and herd size. They were also used to categorize herds into seven herd types and 18 herd sub-type production system categories, based on work conducted previously for herd classification in 2017 [25]. The categories consisted of those of Brock et al. [25] plus “seasonal” herds with data for May and September, but not January, of the classification year, and “unknown” for herds where the data showed that they were active but there were insufficient data to classify them. The herd type categories are listed in the legends to Figs. 8 and 9.

#### Modelled badger abundance

We used the mapped output from a previous study [26], which estimates probability of the occurrence of a badger social group (strictly a main sett), at 100 m resolution, as a proxy for badger abundance.

#### Digital elevation model

Altitude was estimated using a digital elevation model (DEM) calculated at the herd’s centroid (the point identifying its location). (Source: https://www.mapsforeurope.org/datasets/euro-dem).

#### Precipitation

Mean annual precipitation, 2015–2019, in millimeters, at 1 km grid resolution, were obtained from Met Éireann, the Irish Weather Forecast Service (https://www.met.ie/monthly-rainfall-and-temperature-grids/). In our analyses we generally refer to this as rainfall, as snow and other forms of precipitation are rare in the Burren, and in Ireland as a whole.

#### Commonage

A commonage (common land used for grazing) map was obtained for the whole of Ireland, published by the Department of Housing, Local Government and Heritage (https://data.gov.ie/dataset/commonage-gis-dataset).

#### Corinne landcover

Land cover was obtained from the Corinne Programme (https://land.copernicus.eu/pan-european/corine-land-cover) [27]), .

#### Badger control data

We obtained badger control data from DAFM, listing, for each badger sett known to DAFM, the number of restraints (traps) put in place, and the number of badgers removed, through culling, in them, as well as whether it was a main or outlying set (the latter being an additional sett used by the same social group as the main sett).

### Time series and maps

We wanted to examine whether the perceived high bTB risk for the Burren was a real phenomenon, whether it was recent or long-term, and whether it was restricted to the study area or extended beyond it. To this end, we produced an annual time series of the percentage of herds with > = 1 standard CITT reactors for each of four zones, defined with reference to the location of the home farm of each herd: herds within the study area, herds located outside of the study area but less than 5 km from it, herds located between 5 and 10 km of the study area, and herds > 10 km from the study area. Although the rest of our analyses described below used data only up to 2019, as this was the last complete year before our study was initiated and we were able to obtain data for all the data sources up to this year, we calculated the time series from 2005 up to 2022 as, by the time the study was completed, we had bTB testing data up to that point.

To identify any spatial patterns of bTB prevalence within the Burren, we produced annual 5 km resolution gridded maps of the percentage of herds with > = 1 standard reactors, 2005–2019. Furthermore, to disentangle the potential contributors to high bTB prevalence, we produced 5 km resolution grids summarizing a range of variables, for comparison with the bTB prevalence maps. We arranged these into four main groups:

1. Firstly we mapped the physical geography of the area, producing maps of landcover (from the Corine landcover data), precipitation and altitude.
2. We examined the characteristics of cattle husbandry, based on the cattle movement data from 2019. We calculated the density, per square km of land area, of herds with any animals on January 1st, May 1^st,^ or September 1st, measured in separate maps with reference to (i) whether the geographic coordinates for the home farm were located in each grid square or (ii) whether the herd had any land area within the square (based on the LPIS data). Also included in this group of maps were the following, mapped with reference to the geographic coordinates for the home farm: the density of animals per square km, the average number of animals per herd, the in-strength (number of moves into the herd), in-degree (number of herds trading in), in-strength per animal in the herd, the location of common land and the dominant (i.e. highest percentage) herd type.
3. We also mapped the proportion of each seven main herd types and five beef herd sub-types that were present in each square.
4. Lastly, we looked at badger populations and the badger control programme. We mapped the main and outlying badger setts in the area alongside the predicted probability of the occurrence of a badger social group. We also mapped the number of badgers caught, the number of restraints put in place and, as a measure of trapping efficiency, the numbers of badgers caught per restraint, for each sett between 2005 and 2019. As there were fewer data governance constraints with the badger data, we were able to show the raw data (locations of setts, for example) alongside the 5 km gridded summaries.

### Bivariable plots

We used bar charts to plot the characteristics of bTB restricted and not restricted Burren study herds and herds outside the Burren, during the period 2015–2019. We restricted our analyses to the Burren study herds which contained animals on the 1st of January, May and September, 2015–2019, as herd composition on these dates were used to calculate herd size, and we also wanted to select herds which had been continually active during the period.

The variables used for these plots mostly matched those that were used in the time series and maps described above. However, because the analyses were conducted at herd-level, rather than area-based, there were some variables which were omitted, such as land cover, and some which were modified to suit this distinction. The full list of variables comprised: herd type and sub-type, herd size, in-strength, in-degree, moves per animal, maximum and mean value of badger suitability model (calculated across the herd’s land parcels), rainfall (precipitation) and altitude at the herd location, plus the following badger values measured per square km within 2.5 km of the location of the home farm: number of setts, main setts, restraints put in place, badgers caught and badgers per restraint. We also looked at whether the herd had any land parcels outside ( > = 10 km and > = 1 km) the Burren, based on LPIS data, to test whether within-herd movement of animals from outside the area might be associated with increased rates of bTB infection; in this case we restricted the analysis to the two Burren groups (with bTB/ no bTB).

We calculated the proportion of Burren study herds within each of the production system categories and sub-categories, and, within each of these categories, the proportion which experienced a bTB restriction between 2015 and 2019. We plotted this alongside equivalent information for Irish herds which were not in the Burren study population.

For the other variables we showed summary data for four herd groupings, with reference to whether these herds were or were not in the Burren and to whether or not they experienced a bTB restriction at any time between 2015 and 2019. There were therefore four groups: Burren herds with bTB, Burren herds without bTB, outside (i.e. non-Burren) herds with bTB, outside herds without bTB.

**Fig. 2:**
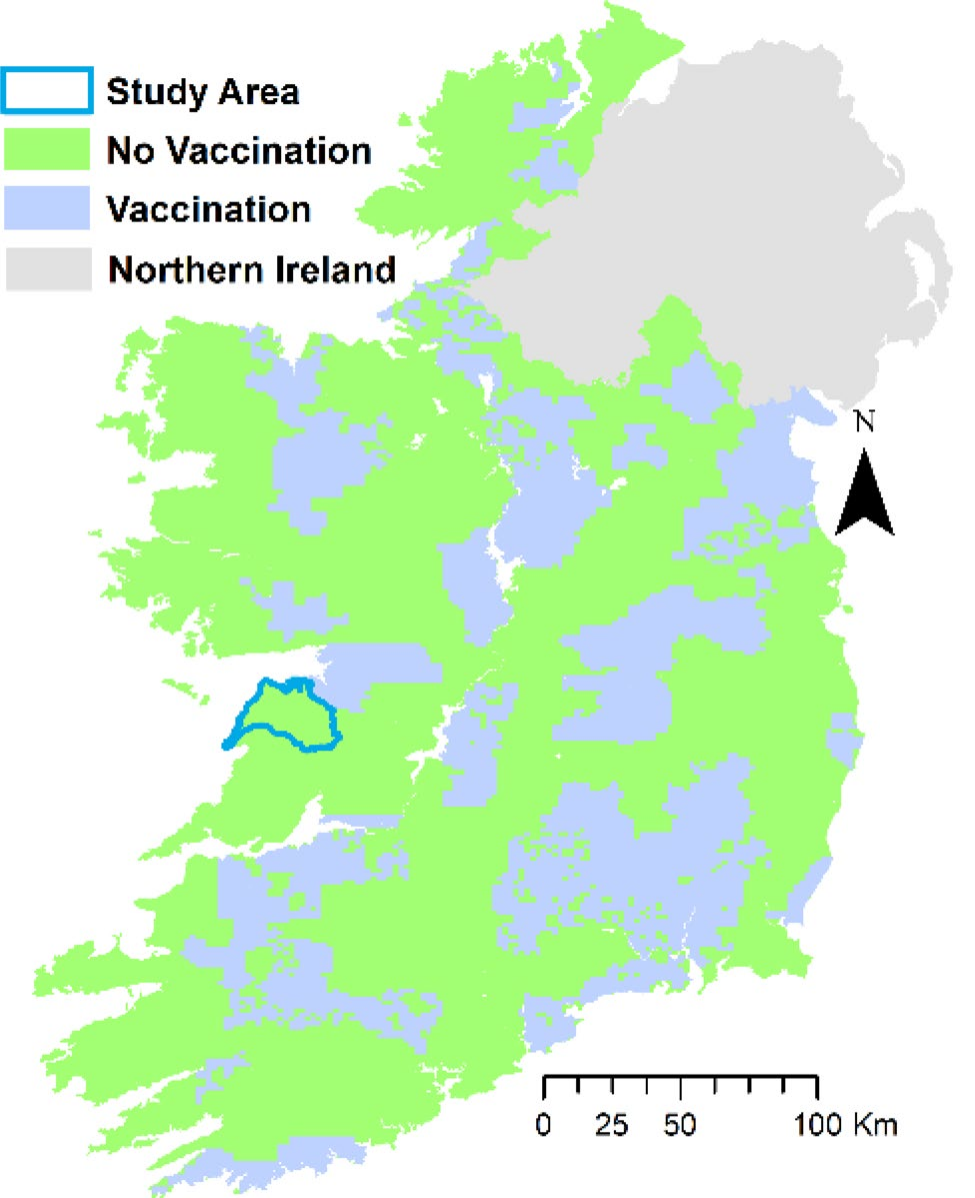
Map of Ireland, including the study area and areas designed for implementation of badger vaccination

For plots based on the badger capture (i.e. removal through culling) records, we excluded areas which had been designated for badger vaccination at any time, so that these plots could be used to compare badger control effort across groups; these areas comprised 30.2% of Ireland but only 0.007% of the study area (Fig. 2). The movement variables were based on yearly averages (2015–2019). In cases where herd type differed between the five years (2015–2019), herd type was assigned randomly from one of the types recorded for the herd during the period.

We also calculated the proportion of skin reactors in the study area with visible lesions at slaughter, 2015–2019, and compared this with herds located outside the study area during the same period.

### Logistic regression

Using the *glm* function in R 4.1.1 (https://www.r-project.org/), binary logistic regression was used to identify risk factors for bTB in the Burren, using Burren herds only, with the dependent variable being a classification of herds into those which had and those which had not experienced the start of a bTB restriction at some time between 2015 and 2019. Herds already in a bTB restriction at the start of 2015 which did not commence a further bTB restriction before 31 December 2019 were excluded from the analysis. Only one of these herds was under restriction throughout this 5 year period. For the same reasons as for the bivariable plots, we again restricted our analyses to the Burren study herds which contained animals on the 1st of January, May and September, 2015–2019.

Previous analyses have identified herd type and size as important risk factors for bTB [23]. Also, herd size and type might be expected to exhibit co-linearity with other variables we were interested in, especially cattle movement. In addition, because our study population (herds in the Burren) was quite small we wanted to keep the number of explanatory variables to a minimum. With these considerations in mind, we used a case-control study design and developed a model of herd-level bTB where bTB positive herds (cases) were matched with bTB free herds (controls) on the basis of herd sub-type and size, which allowed us to explore more parsimonious models and account for unmeasured confounders. This was done using the python programming language using an iterative process: all herds in the Burren study area which experienced the start of a bTB restriction at any time between 2015 and 2019 were ordered randomly, and, in cases where multiple bTB restrictions occurred for the same herd, one of these restrictions was randomly selected. Then each herd was in turn matched to another Burren herd of the same herd sub-type which was as close as possible in herd size, but which did not experience the start of a bTB restriction between 2015 and 2019. This herd size matching was alternated such that if a restricted herd was matched to a non-restricted herd which was larger than it, the next herd would be matched to one which was smaller. This process was done without replacement – as soon as a herd had been matched once, both it and the matching herd were removed from the matching process. This whole process was done for 1000 iterations, with the order in which the herds were processed arranged randomly. In each iteration, a small but variable number of herds could not be matched, and the final iteration selected was the one that gave the lowest product of the average difference in herd size and the number of herds which could not be matched. A 1:1 ratio (as opposed to a 1:2 or 1:3 ratio) was used for cases and controls, because this allowed us to ensure that the maximum number of cases could be matched to a control and that differences in herd size between cases and controls was minimized.

Nine variables were considered for inclusion in the model.

Four of these were movement variables, based on findings in [23, 28, 29] on the relationship between inward movements and bTB risk in UK and Irish herds. Each of the movement variable was calculated with reference to movements into the herds within a two year period prior to the start of the herd restriction (for each herd which did not experience a bTB restriction, the restriction date of the restricted herd to which it was matched was used). One of these variables was in-strength (the number of animals moved into the herd). The remaining three counted the number of distinct herds that incoming animals came from. In-degree counts all herds that incoming animals came from. The other two variables only counted herds which themselves had at some time experienced a bTB restriction –in one case this was limited to bTB restrictions that started within a five year period prior to each move (*in-degree bTB 5 years*) and in the other case herd restrictions within two years after the move were also included (*in-degree bTB 7 years*).

Other variables considered for inclusion in the model were mean annual rainfall (precipitation), altitude at the herd location, the maximum value for modelled badger abundance across the farm’s land holdings (which we will refer to as the “badger metric”), all three based on findings in [23], and two binary variables measuring whether the farm owned any parcels of land > 1 Km or > 10 Km, respectively, from the study area.

A forward stepwise approach was used for the modelling. First, bivariable regressions were used to reject those variables which did not result in a lower Akaike Information Criterion (AIC) [30] than the null model. The variable which brought about the largest reduction in AIC was then used as the basis for a multivariable model, with the remaining variables added successively in order to select the next one for inclusion in the model, again on the basis of the AIC. This process was repeated until no further variables could be added to the model without bringing about an increase in the AIC.

Goodness of fit of the model was tested using the Hosmer-Lemeshow test using 10 groups [31], as well as an examination of the receiver operating characteristic (ROC) curve. We examined whether multicollinearity was an issue in the models using Spearman correlations and through an examination of the Variance Inflation Factor (VIF). We did these tests using the Performance Analytics Package of R (https://github.com/braverock/PerformanceAnalytics; https://www.rdocumentation.org/packages/PerformanceAnalytics/versions/2.0.4/topics/PerformanceAnalytics-package).

When presenting odds ratios and confidence intervals of any continuous variables selected in the final model, we standardised them by rerunning the model after dividing each variable by its standard deviation, to enable readier comparison of their effects.

## Results

1,038 herds with at least one bTB test between 2015 and 2019 had land within our study area. From this population, 939 (90%) herds fulfilled the criteria for our study population for the mapping and bivariable analyses, of which 253 (27%) experienced at least one standard reactor between 2015 and 2019.

### Time series and maps

**Fig. 3:**
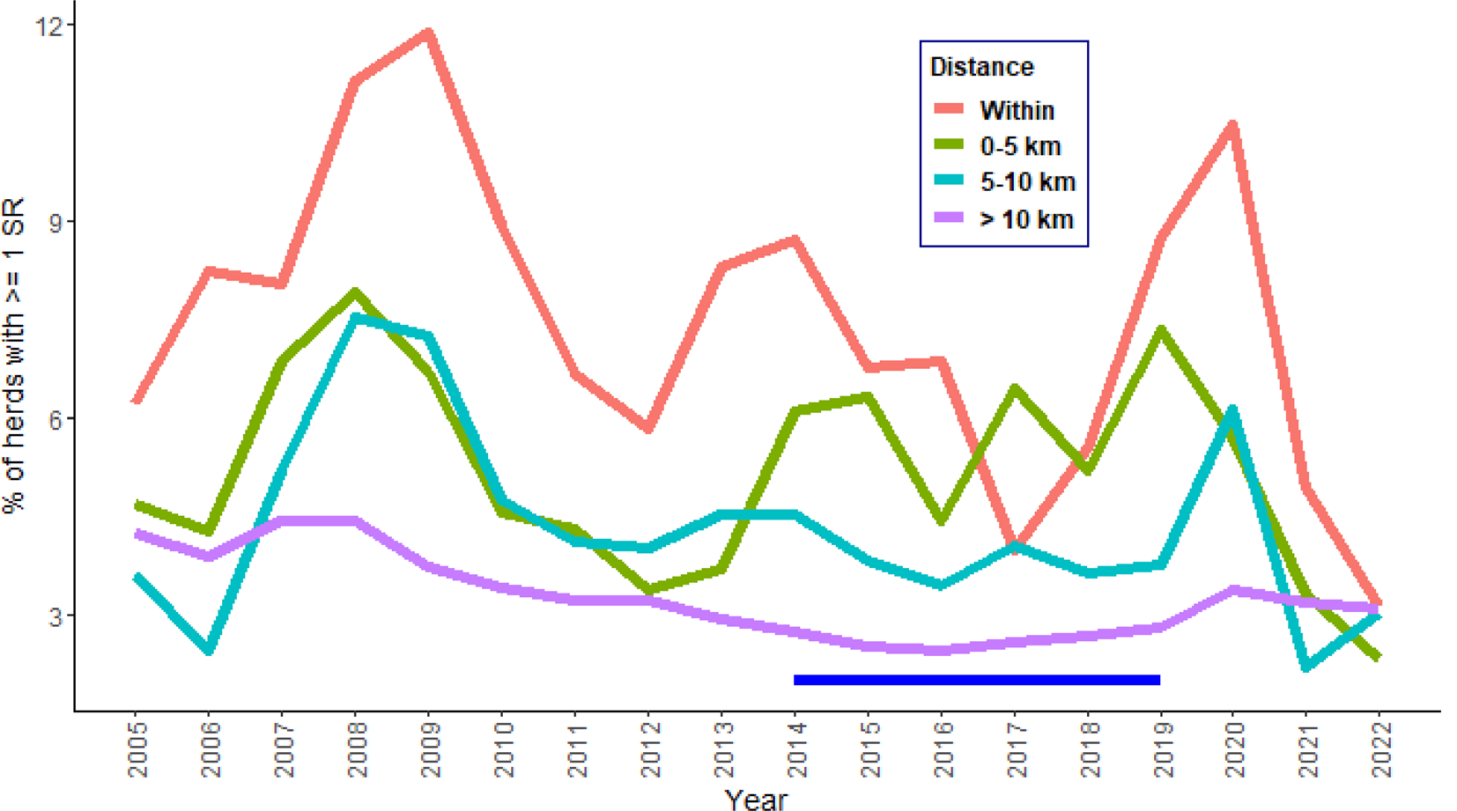
Percentage of herds in the study area with one or more standard reactors in each year, 2005–2022, including herds within the study area, herds located outside of the study area but less than 5 km from it, herds located between 5 and 10 km of the study area, and herds > 10 km from the study area. The study period for the multivariable analyses, 2015-19, is indicated using the horizontal blue line

Herds within the study area (pink line, Fig. 3) showed higher bTB prevalence (≥ 1 CITT standard reactor) when compared to Ireland as a whole and when compared to areas outside the study area but in close proximity to it. This pattern went back at least as far as 2005. In the period after the current study (that is, post 2019), bTB prevalence in areas in the study area decreased substantially and by 2022 were similar to those outside the study area (Fig. 3).

**Fig. 4:**
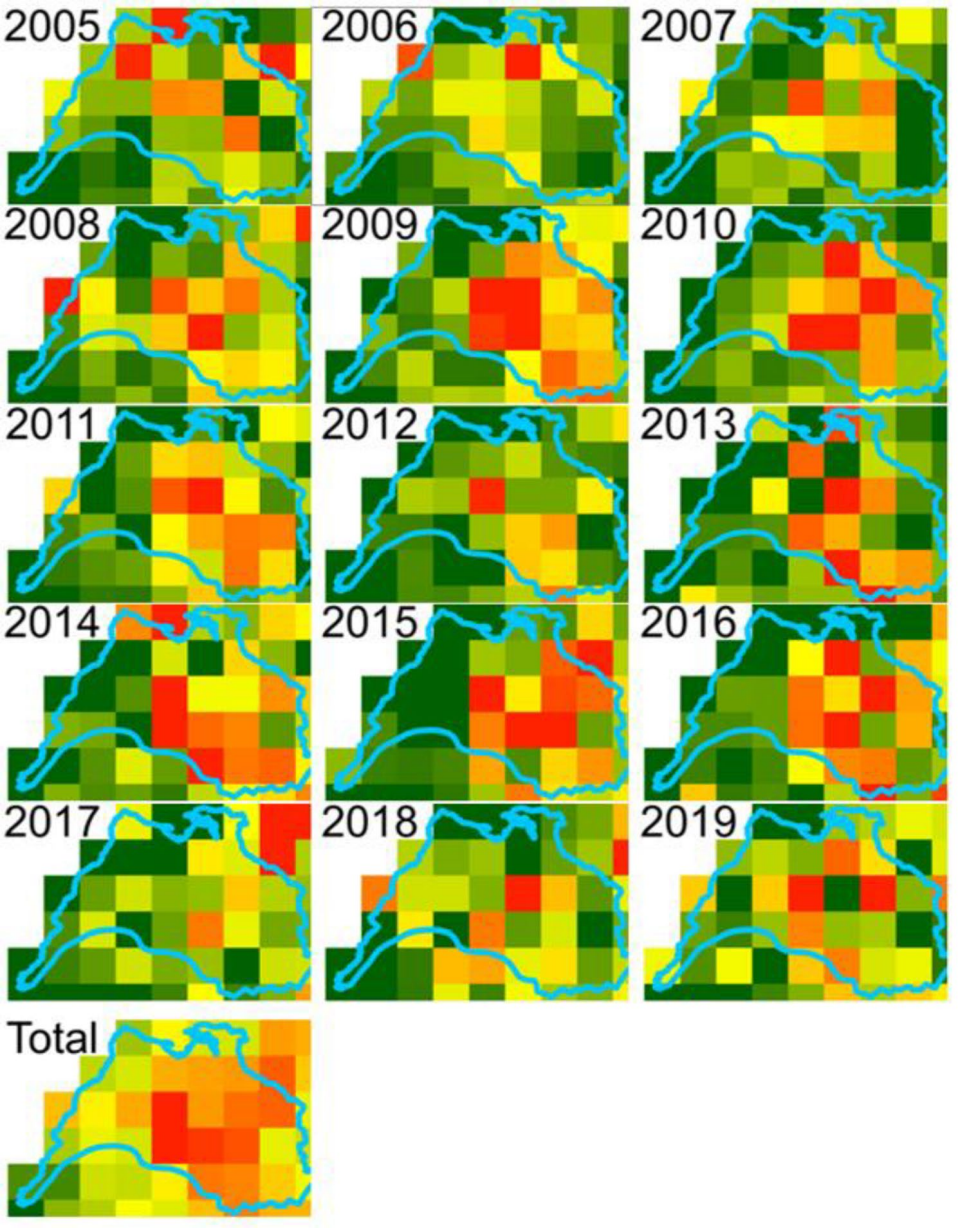
The percentage of herds with one or more standard reactors in each year, 2005–2019 aggregated into 5 km grid squares, and, bottom left, the mean % of herds aggregated over the entire 14-year period

**Fig. 5:**
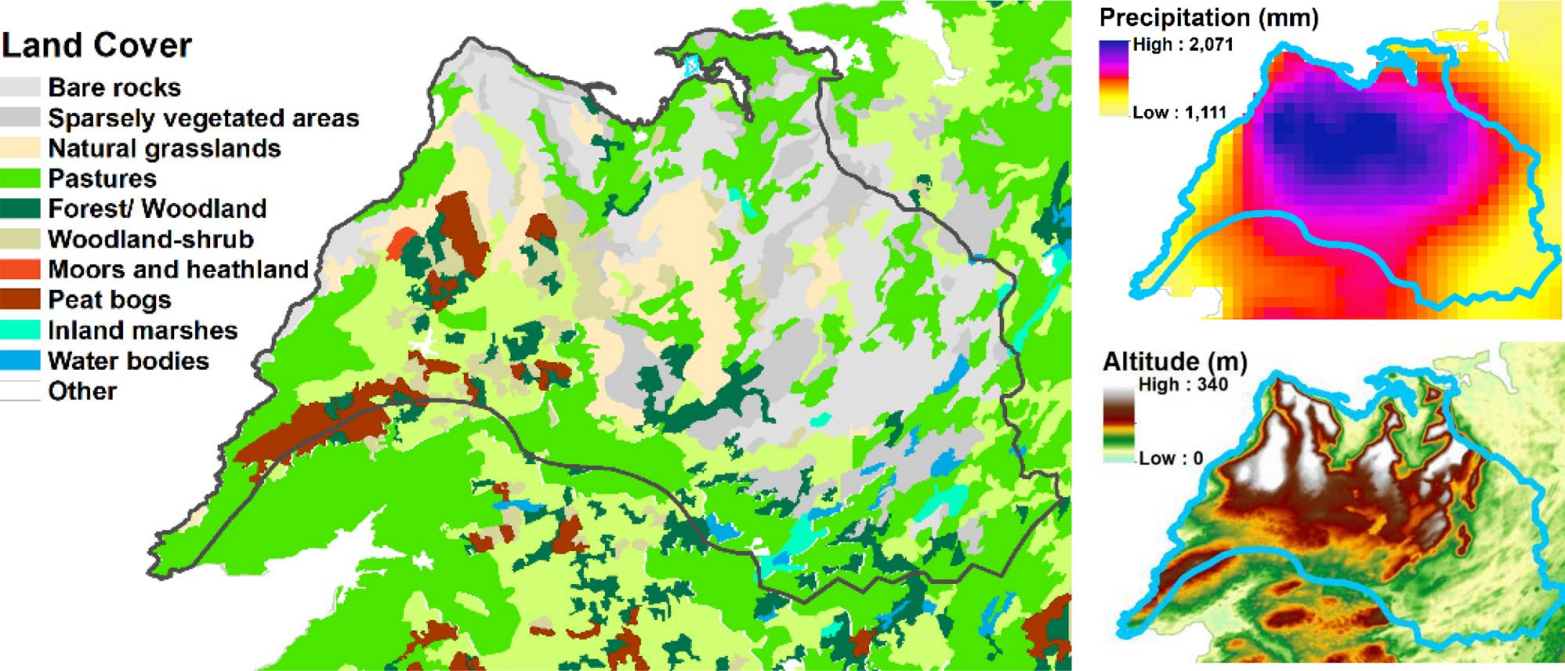
Landcover, precipitation and altitude in the study area, which is delineated with a black line on the land cover map and a blue line elsewhere

**Fig. 6:**
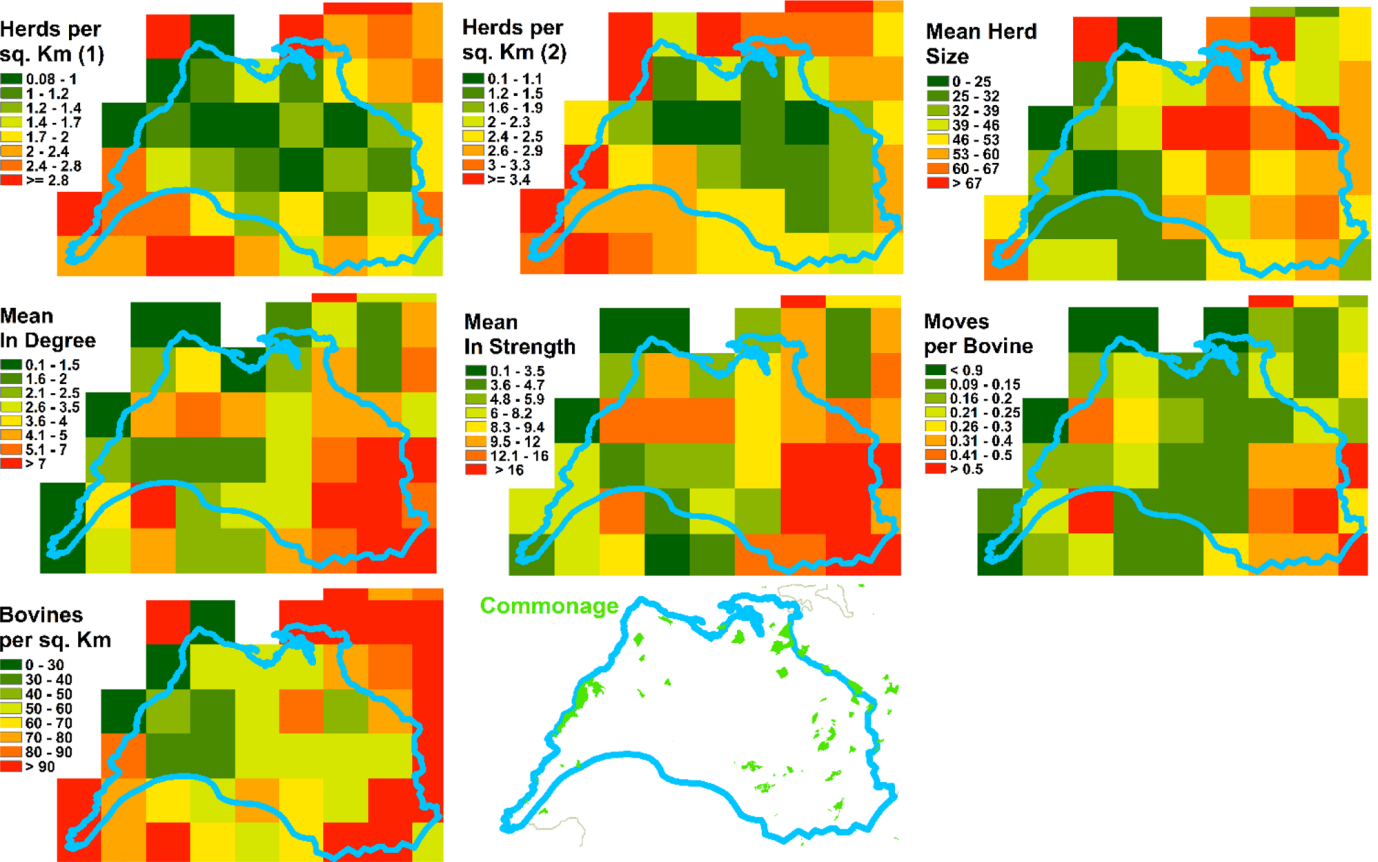
Cattle herds per square km measured with reference to the location of the herds’ geographic coordinate centroid (Herd per sq. km (1)), Cattle herds per square km measure measured with reference to the location of the herds’ land parcels (Herd per sq. km (2)), Herd Size, In degree, In strength, (inward) Moves per bovine, Bovines per square km and the location of commonage. Geographic coordinate centroids were used for the location of herds for all the gridded maps with the exception of Herds per sq. km (2)

An examination of the spatial pattern of bTB prevalence showed that there was no single hotspot in the Burren but that areas of high prevalence tended to differ from year to year, although overall reactor rates were highest towards the north east of the study area (Fig. 4), which broadly corresponds to the area of highest elevation and bare karst geology with high levels of rainfall, but not with high levels of commonage, herd and cattle density or cattle movement (Figs. 5 and 6). There was some evidence that herds tended to be quite large in parts of this high prevalence area (Fig. 6, mean herd size).

**Fig. 7:**
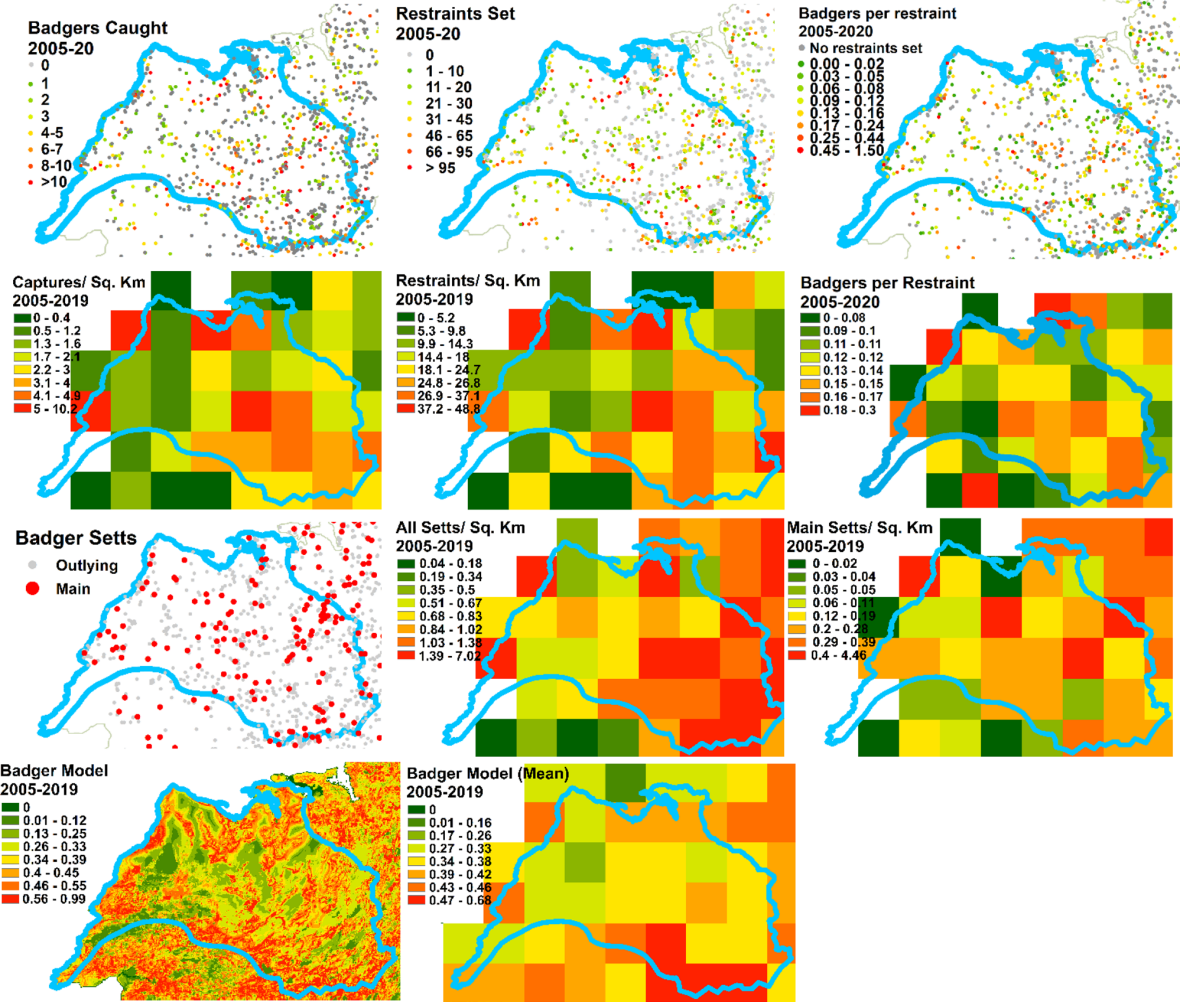
Badger data within and around the study area. The following are mapped at sett locations and as mean per square km values at 5 km grid resolution (rows one to three), based on 2005–2019 data: number of badgers caught, the number of restraints set, the numbers of badgers caught per restraint (as a measure of trapping efficiency), the number of badgers caught and restraints per square km, the number of badgers caught per restraint, the number of badger setts (all setts, main setts). The outputs of the badger abundance model (Byrne et al., 2014) are also presented (bottom row), at the original, 100 m resolution that the model was generated at and as mean values at 5 km resolution

There were no strong spatial patterns in any of the badger data, although there was a tendency for more restraints to be set and more badgers culled towards the east of the study area, possibly due to more capture effort being expended on areas with high herd prevalence (Fig. 7). The number of badgers caught per restraint was fairly uniform across the study area. The badger model tended to predict greater probability of badger social groups toward the southeast of the study area.

**Fig. 8:**
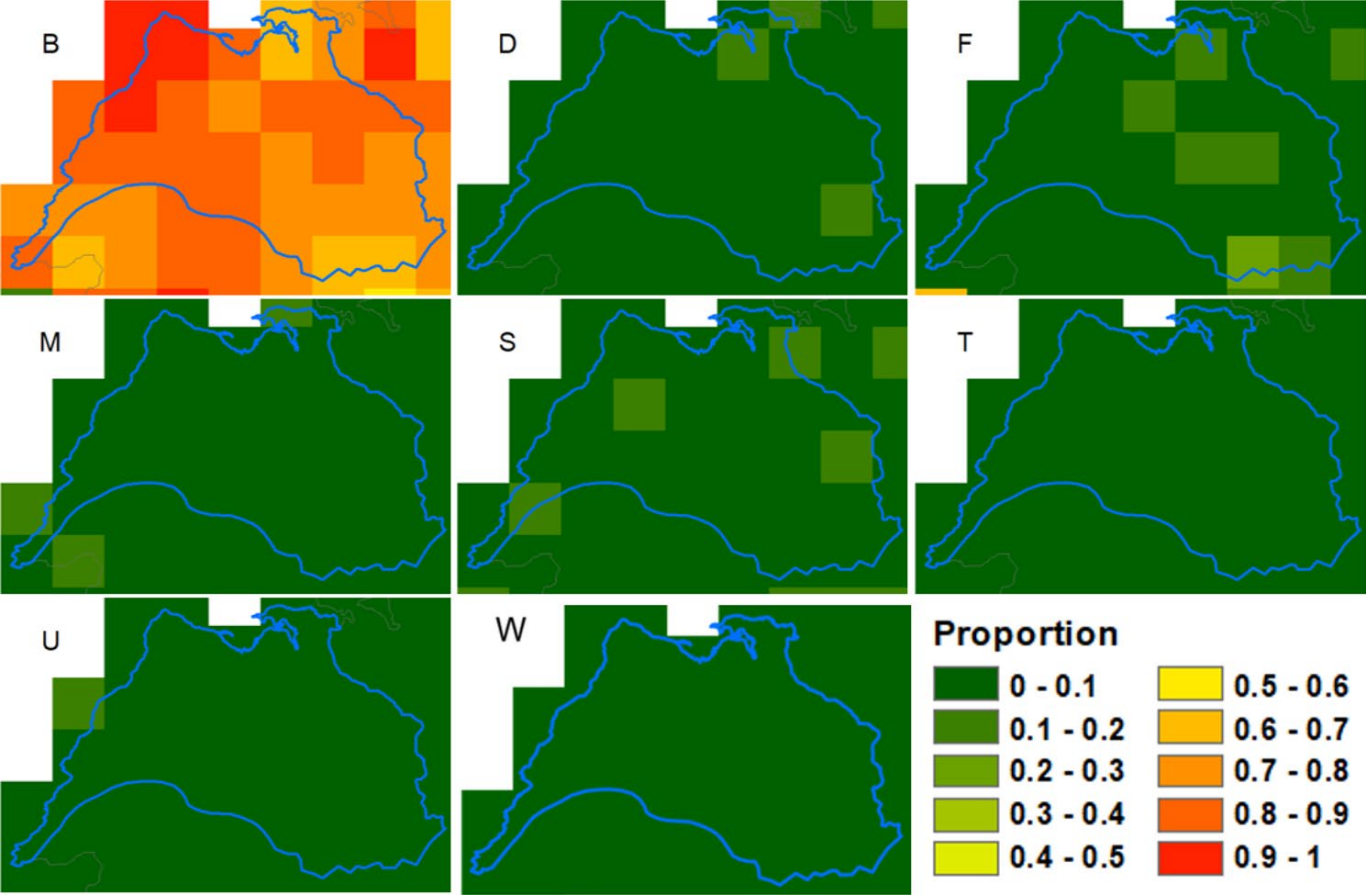
Proportion of herds in the study area that were of each main production type, based on the classification method of Brock et al. (2021). B = Beef, D = Dairy, F = Fattener, M = Mixed, S = Stores, T = Trader (Dealer), U = Unclassified; W = Seasonal

**Fig. 9:**
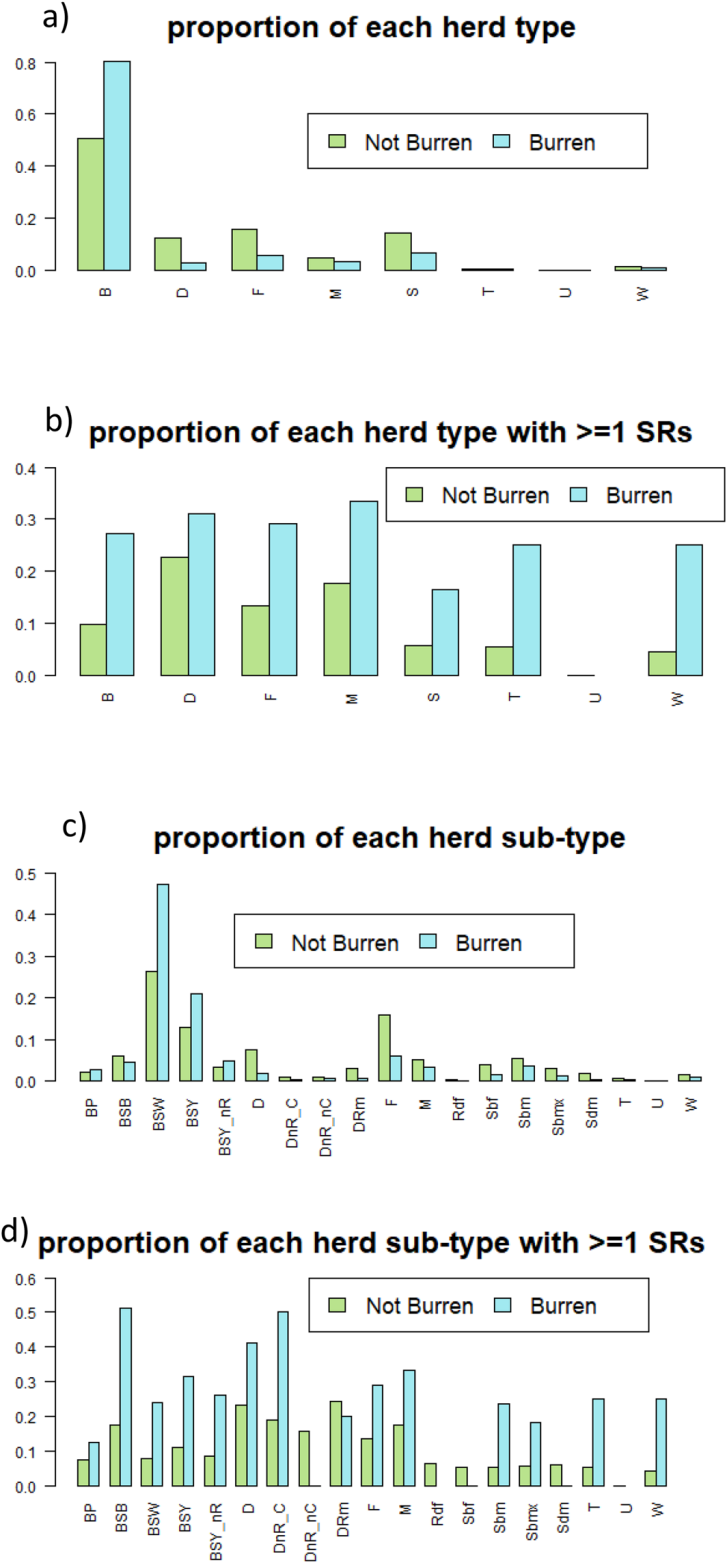
Proportion of all herd types (**a**) and herd subtypes (**c**) in the study area (Burren) and outside the study area (Not Burren), and the proportion within each herd type (**b**) and subtype (**d**) reporting one or more standard reactors between 2015 and 2019, based on the classification of Brock (2021). See Fig. 8 for main herd types; sub-types are as follows: BP = Beef Pedigree, BSB = Beef Suckler to Beef; BSW = Beef Suckler to Weanling; BSY = Beef Sucker to Youngstock; BSY_nR = Beef Suckler to Youngstock non-rearing; D = Dairy (standard production); DnR_C = Dairy non-rearing, using contract rearing, DnR_nC = Dairy non-rearing, not contract; DRm = Dairy, rearing males; F = Fattener; M = Mixed; Rdf = Rearing Dairy Females; Sbf = Stores for beef females; Sbm = Stores for beef males; Sbmx = Stores for mixed sex; Sdm = Stores for dairy males; T = Trader; U = Unclassified; W = Seasonal

**Fig. 10:**
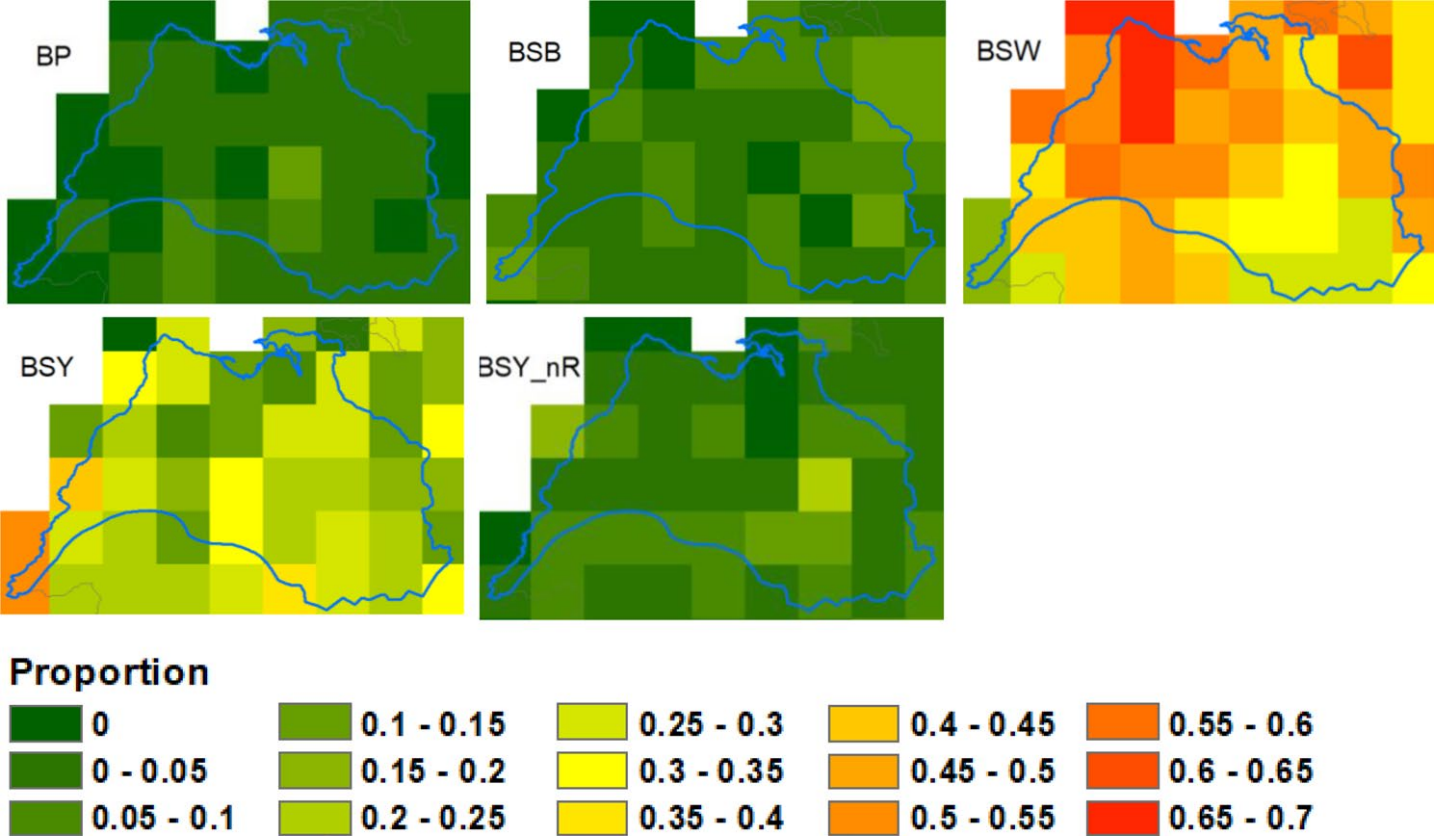
Proportion of each beef herd subtype in the study area, based on the classification method of Brock et al. (2021). See Fig. 9 for a description of each subtype

Approximately 80% of herds in the study area were of beef production type, i.e., producing their own beef calves, compared with about 50% across Ireland (Figs. 8 and 9). Most of these beef herds were of the suckler to weanling or suckler to youngstock types (Figs. 9 and 10). Concomitantly, a much smaller proportion of herds were of dairy, fattener (finishing animals prior to slaughter) and stores (raising, but not producing, cattle) types. All the herd types were more likely to have a CITT test reactor in the Burren than nationally and this was particularly evident for beef, stores, trader and seasonal herds, although it should be noted that very few herds in the study area were of the latter two groups. There was less variability in the proportion of bTB positive herds across the various herd types in the Burren compared to nationally (Fig. 9).

### Bivariable plots

**Fig. 11:**
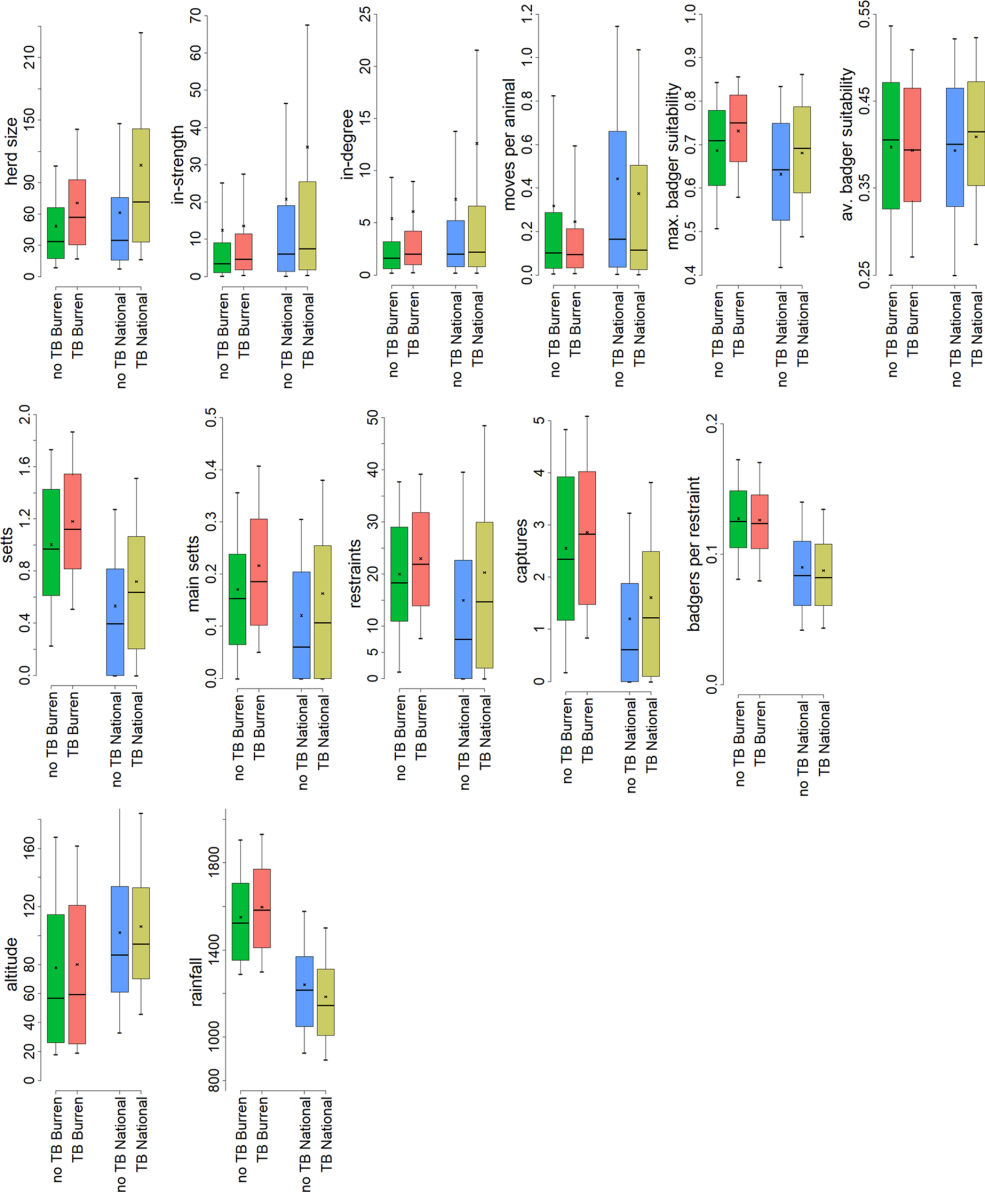
Thirteen indicators summarised with reference to four groupings of Irish herds – study area herds with no standard reactors, 2015–2019 (no TB Burren, green bars), study area herds with at least one standard reactor, 2015–2019 (TB Burren, red bars), herds outside the study area with no standard reactors, 2015–2019 (no TB National, blue bars), herds outside the study area with no at least one standard reactors, 2015–2019 (TB National, mustard bars). See text for a description of each indicator. Each bar shows median, lower and upper quartile, tenth and ninetieth percentile and the mean

In the bivariable plots, it was apparent that in the Burren bTB herds tended to be larger than non-bTB herds, which matches the national pattern; however, Burren herds in both groups were on average smaller than herds outside the Burren (Fig. 11). Similarly for the movement data, in-degree and in-strength, bTB herds tended to have more movement than non-bTB herds. This phenomenon was less pronounced within than outside the Burren; in any case Burren herds as a whole experience fewer movements than other herds. Interestingly, non-bTB herds showed a higher number of inward moves per animal than the bTB herds, both within the Burren and nationally (Fig. 11).

The predicted probability of the presence of a badger social group was slightly higher for bTB herds both in the Burren and nationally (Fig. 11). This was more pronounced when the maximum predicted probability within the farm rather than the mean, was used. Similarly more setts (total and main setts) were located in proximity to the bTB herds both in the Burren and elsewhere, with the Burren having a larger number of setts than the average elsewhere. The number of restraints put in place and the number of badgers culled were higher for bTB herds in the Burren than for non-bTB herds; badgers per restraint were about the same for both groups. Median values for all three of these measures were higher in the Burren than elsewhere (Fig. 11).

Ninety-three herds had some land in the study area and some land over 10 km from it. Of these, 86 had bTB test data for 2015–2019, and were therefore included in our analysis. The probability of herd restriction during that time was almost identical for this group of herds and those with no land over 10 km away (0.2696 versus 0.2674). Equivalent figures for over 1 km were 347 herds of which 326 were included in our study population. For these herds, probability of herd restriction was somewhat higher than for those with no land over 1 km away (0.2529 versus 0.3006).

The percentage of skin reactors with visible lesions at slaughter in the Burren compared to elsewhere was very similar – 32.9% versus 32.3%.

### Logistic regression

For the logistic regression 260 herds which started a bTB restriction between 2015 and 2019 were matched on herd type and herd size with 260 herds which did not experience a breakdown. The mean difference in herd size between matched herds with and without a bTB breakdown (Herd with breakdown – Herd no breakdown) was small: 0.034 animals. Bivariate models with herd type and herd size as the explanatory variables had no effect versus the null deviance, indicating that the matching had worked effectively.

In the bivariable models, only the rainfall variable resulted in a significant improvement on the null model, measured as the difference between the residual and null deviance using a chi-squared test. Of the remaining variables, only altitude produced a better fitting model, measured by the AIC. Adding the *in-degree bTB 7 years* variable produced a slightly lower AIC and further adding the *badger metric* variable produced a slight further improvement (Table 1).

**Table 1:** Logistic regression model for the occurrence of a new bTB breakdown, 2015–2019, in a sample of Burren cattle herds (*n* = 520). For continuous variables Odds Ratios and CLs have been standardised by rerunning the model after dividing each variable by its standard deviation. AIC: Akaike Information Criterion. CL = 95% Confidence Limits; StDev = Standard Deviation of the Variable; St Odds Ratio = Standardised Odds Ratio. Change in AIC shows the increase in the AIC of the model if the variable is removed from the final model. Rainfall and altitude are calculated for the location of the home farm of the herd. In-degree bTB 7 years is a measure of the number of farms the herd bought cattle from in the two years before the breakdown, where these selling herds themselves experienced a breakdown during period five years before or two years after the animals were sold. Badger metric is a measure of predicted badger abundance based on Byrne et al., 2014. These variables are described in detail in the Methods section

	Coefficient	Standard Error	StDev	St. Odds Ratio (+/- 95% CL)	*P*	AIC Change
Intercept	-3.54442	0.93548			< 0.01	
Rainfall (mm)	0.00189	0.00054	223.38	1.53 (1.21–1.94)	< 0.01	10.98
Altitude (m)	-0.00395	0.00197	60.62	0.79 (0.62–0.99)	0.045	2.09
In-degree bTB 7 years	0.0114	0.00905	14.87	1.18 (0.96–1.67)	0.207	0.43
Badger Metric	1.16842	0.79391	0.12	1.14 (0.96–1.37)	0.141	0.18

**HL** : 7.34 *p* = 0.50 **AIC**: 712.85(model with intercept only: 720.87) **Area under ROC Curve**: 0.60

Spearman correlation, ρ, between the rainfall and altitude variable was 0.61. Correlation between the other explanatory variables in the model varied between − 0.09 (*badger metric* with *altitude*) and 0.07 (*badger metric* with *in-degree bTB 7 years*). The Hosmer-Lemeshow test was not significant (*p* = 0.50) and the area under the ROC curve was 0.6. The VIF for the variables in the final model showed that multicollinearity was not a major issue, with no VIF values higher than 1.6.

**Discussion**.

Our results suggest that in most respects Burren herds would be expected to be at low risk of bTB infection. The predominant herd type in our study population was beef, a type which has found to be relatively low risk for bTB in Ireland and the UK [23, 28, 32]. Within this group the predominant sub-type was suckler to weanling, representing almost half of the herds in the study area, which this study has shown to be relatively low bTB risk nationally (Figs. 9 and 10). Herd sizes were generally smaller than the national average and inward movements, whether measured by in-strength, in-degree or in-strength per animal, were also lower than average (Fig. 11), whereas both these factors have been shown to be positively associated with bTB risk in Ireland [23, 29]. The altitude where a herd is located has been found to be positively associated with bTB risk [23], whereas Burren herds are on average located at lower altitudes than the national mean (Fig. 11).

Furthermore, we found no evidence that badger control (i.e. removal through culling) is particularly poor or that badgers are difficult to capture in the Burren. There are on average more badger setts located within 2.5 km of herds than the average nationally, but this may in part be due to more search effort being expended in the Burren, with its frequent history of bTB breakdown, than elsewhere (Fig. 11).

As described in the methods section, we modelled badger abundance using two metrics based on a previous badger abundance model [26], calculated at 100 m resolution. One of these metrics used the maximum value, and the other the mean value, from those 100 m grid squares located within land parcels occupied by the herd. Using the former metric (maximum value), higher badger abundance (strictly speaking, higher probability of a badger social group) was predicted for Burren herds with a history of bTB in comparison with other herds in the Burren or for both groups (bTB/non-BTB) outside the Burren. However, this pattern was not so evident when looking at the metric calculated using the mean value (Fig. 11). A previous study [12], based on answers to a questionnaire from Burren herd owners, found that previous bTB breakdown for a herd was associated with the percentage of land which was inaccessible to those attempting to locate badger setts; however, our study shows that the number of total setts and main setts located, restraints set, and captures were higher for bTB herds in the Burren than for non-bTB herds. Badgers per restraint were about the same for both groups.

Having land outside the Burren did not appear to be an important risk factor. Herds with land over 10 km from the study area had about the same risk as those without this characteristic and, although having land over 1 km did occur slightly riskier (Fig. 11), neither of these characteristics merited inclusion in the final regression model, which suggests than other factors taken account of in the model are likely to explain these differences.

Rainfall was relatively high for all Burren herds, and higher for those with a history of bTB (Fig. 11). This echoes a previous study on cattle herds in County Wicklow, in the east of Ireland, which found that rainfall was positively correlated with herd-level bTB occurrence in a study of 550 herds in the west of the county [33]. It also supports the finding of a previous study [34] that temperature and moisture related climatic indicators were important predictors of bTB incidence in cattle herds in England and Wales. This positive association with rainfall was confirmed in the binary regression analysis – rainfall was the only one of the candidate explanatory variables which produced an improved fit over the null model in the bivariable analysis, and the standardised odds ratio for rainfall gave a 53% increase in the odds of herd breakdown for each standard deviation (223 mm) increase in rainfall (Table 1); Fig. 5 shows that this would be about one fifth higher than the rainfall experienced by herds in the driest areas and 11% higher for those in the wettest. Rainfall is relatively high across the Burren as a whole, compared to other areas of Ireland, and, furthermore, it is generally highest in areas of the Burren where bTB rates have been highest. As herd size tended to be larger for Burren herds which were positive for bTB than for negative herds, one possible explanation is that the process driving bTB prevalence in the Burren is the effect of harsh winter conditions driving large cattle herds to seek shelter together. This combined with greater potential contact with bTB infected wildlife, deteriorating feed quality and the effects of exposure to harsh weather, may explain why bTB prevalence has been so high. Survivorship of *M. bovis* in the environment may also be higher in damper areas with less exposure to sunlight [35]. The fact that the slaughterhouse lesion rate was similar for skin-test positive animals in the study area compared with those outside it suggest that test-specific effects, such as decreased test specificity due to exposure to mycobacteria spp. in the environment [36], is unlikely to be an import factor.

The final regression model, which was produced by adding further variables, in addition to rainfall, also included altitude, in-degree with bTB, and badger abundance. However rainfall was by far the most important variable in this model, when measured by the standardised odds ratio, change in AIC from a model with this variable omitted, and the significance level. It should be borne in mind that altitude was quite strongly correlated with rainfall (ρ = 0.61) and carried a negative coefficient with bTB infection in the final model, whereas it carried a positive coefficient in the equivalent bivariable model, as it had in national level bTB models produced in a previous study [23]. This suggests that, when the effects of rainfall are taken into account, lower altitude increases the risk of bTB infection in Burren herds.

The mapping outputs allowed us to examine whether the spatial distribution of any of the variables matched that of the incidence of positive bTB skin tests (Figs. 4, 5, 6, 7, 8 and 10), where elevated risk could be seen in herds to the northeast and central areas of the study areas (Fig. 4). However, most of these variables showed no clear relationship with this pattern – inward movements and herd density were not particularly high in these areas, high risk herd types (such as dairy and fattener herds) were not found there in great numbers, and modelled badger abundance and badger control operations appeared to operate as effectively in these areas as elsewhere. Exceptions to this were altitude, rainfall, and mean herd size, for which elevated values showed some correspondence with areas of high bTB incidence (compare Fig. 4 with Figs. 5 and 6). The area of elevated land in the north of study area was matched by elevated rainfall in this area, which was confirmed by the strong correlation between the two mentioned above.

Herd size was also larger in this area relative to herd size in the study area as a whole and this, combined with a known association between herd size and bTB incidence was a reason for us matching on herd size in the regression analysis. We also matched on herd type, partly because the herd types known to be most strongly associated with bTB [23] were not typical of the Burren but also because there were 17 herd types and there was a need for parsimony given the relatively small sample size. It needs to be emphasized that our regression model does not indicate that herd size and type do not play a role, it rather shows those factors that are important once herd size and type have been taken into account.

This analysis covers the period 2005-19, when bTB incidence in the Burren was much higher than for the rest of Ireland considered together (Fig. 3). However, Fig. 3 shows that since 2020 there has been a sharp decline in incidence, and in 2023 rates in the Burren matched those elsewhere. It is beyond the scope of this study to analyse the reasons for this improvement, but, anecdotally, the turnaround may be explained by intensification of interventions by DAFM veterinarians and of badger culling and badger sett identification in the area, epidemiological investigations of breakdowns, bTB test quality control, six monthly testing of the whole Burren area, prompt reactor removals, and very constructive engagement with farmers, veterinarians and the Burren Programme *(Eoin Ryan, pers. comm.)*

A weakness of our study is that we were not able to match our data with the questionnaire study conducted by Clarke et al. [12], as due to confidentiality considerations responses were not matched to individual herds. Whilst a desire for confidentiality on the part of herd owners is understandable, perhaps follow up studies might be done in such a way that herd owners might be convinced to allow their herd numbers to be matched to their survey responses. Survey data matched to the data we used in our analyses, and combined with further fieldwork and the use of data sources which were unavailable for this study, such as liver fluke surveillance records, would allow us to address the possible role of factors which we were unable to address, such as the role of genetic factors, liver fluke infestation and feral goats. Such data would also allow us to better disentangle the contribution of factors such as badger abundance and control, use of housing, quality of feed, and weather. Whole genome sequencing of *M. bovis* in infected wildlife and cattle could also elucidate the roles of intra and inter-herd contacts, as well as wildlife [37].

## Conclusion

In summary, our analysis has revealed that rainfall, or some unmeasured factor which is closely correlated with it, was a significant driver of elevated rates of bTB in the Burren during the period prior to 2020. More detailed studies, where survey responses are matched to information on test results, animal movement, herd size and type, badger control and environmental variability, might allow us to better understand the drivers of elevated bTB incidence in the Burren and elsewhere.

## Data Availability

The datasets generated during and/or analysed during the current study are not publicly available due to GDPR considerations (to protect herd owners).

## Abbreviations

AHCS: Animal Health Computer System
AIC: Akaike Information Criterion
AIM: Animal Identification and Movements
bTB: Bovine tuberculosis
CITT: Comparative Intradermal Tuberculin Test
DAFM: Department for Agriculture, Food, and the Marine
LPIS: Land Parcel Information System
ROC: Receiver Operating Characteristic
SICTT: Single Intradermal Comparative Tuberculin Test
VIF: Variance Inflation Factor
